# Predicting pulmonary function using thoracic deformity parameters in early onset scoliosis patients

**DOI:** 10.1371/journal.pone.0329199

**Published:** 2025-07-31

**Authors:** Mattan R. Orbach, Patrick J. Cahill, Annalise Noelle Larson, Ron El-Hawary, Oscar H. Mayer, Sriram Balasubramanian

**Affiliations:** 1 Sidney Kimmel Medical College, Thomas Jefferson University, Philadelphia, Pennsylvania, United States of America; 2 School of Biomedical Engineering, Science and Health Systems, Drexel University, Philadelphia, Pennsylvania, United States of America; 3 Division of Orthopaedic Surgery, The Children’s Hospital of Philadelphia, Philadelphia, Pennsylvania, United States of America; 4 Department of Orthopedic Surgery, Mayo Clinic, Rochester, Minnesota, United States of America; 5 Department of Orthopedics, Boston Children’s Hospital, Harvard University, Boston, Massachusetts, United States of America; 6 Division of Pulmonary Medicine, The Children’s Hospital of Philadelphia, Philadelphia, Pennsylvania, United States of America; University of Turin: Universita degli Studi di Torino, ITALY

## Abstract

**Introduction:**

Thoracospinal deformities in early onset scoliosis (EOS) patients often lead to thoracic insufficiency syndrome, in which respiration or lung growth is impaired. Pulmonary function tests (PFTs) are used to assess pulmonary deficits but are challenging to comply with for EOS patients, who typically are between 5 and 10 years old. Thus, the objective was to predict PFT values in EOS patients directly from deformity parameters measured on routine radiographs.

**Methods:**

Corresponding preoperative radiographs and PFT values were retrospectively obtained from 47 EOS patients (13M/34F; mean age: 9.8 ± 3.0 years), and 19 literature-based deformity parameters were measured. Multiple linear regression (MLR) analyses using an exhaustive search feature selection method were used to estimate percent predicted forced vital capacity (%FVC) and forced expiratory volume in one second (%FEV_1_). Ten percent of the dataset was set aside to validate the predictive accuracy of the MLR models.

**Results:**

The additive contributions of multiple thoracospinal deformity parameters successfully yielded significant (*p* < 0.001) MLR models that predicted %FVC (R^2^ = 0.54) and %FEV_1_ (R^2^ = 0.59) in EOS patients. For the validation test, no significant differences (*p* > 0.05) in prediction error magnitudes were found.

**Conclusion:**

The developed MLR models provide the highest reported precision for predicting PFT values in EOS patients from radiographic deformity parameters. Additionally, a key subset of deformity parameters was identified, and their relative contributions to predicting PFT values provide quantitative metrics to guide surgical treatment.

## Introduction

Early onset scoliosis (EOS) is a complex, 3D spine and ribcage deformity defined as a lateral spinal curvature ≥ 10 degrees that is diagnosed before 10 years of age, regardless of etiology [1,2]. The thoracospinal deformities in EOS often lead to thoracic insufficiency syndrome, defined as the inability of the thorax to maintain normal respiration or lung growth [3]. Generally, lung volume is contingent upon the width and depth of the ribcage and the height provided by the thoracic spine, and pulmonary function mechanics rely on the diaphragm and secondary respiratory muscles to move the thorax and allow for ventilation. In EOS, these structures are often compromised due to a deformed and limited thoracic volume that can cause chronic restrictive lung disease, a more rigid chest wall, and respiratory muscle impairment [4]. However, the combined effects of altered thoracospinal dimensions on pulmonary function in such deformities are poorly understood.

To monitor the EOS patient population and study the impact of surgical interventions, pulmonary function tests (PFTs) such as spirometry and plethysmography are routinely used to measure lung volumes, airflow, and gas exchange. PFTs are generally done for children starting at five years of age and require the patient’s cooperation and ability to follow instructions in order to perform inspirations and expirations with maximal effort [4]. Although cost-effective and non-invasive, PFT data from young patients can be challenging to collect due to poor patient cooperation [5,6]. In such cases, computed tomography (CT)-based methods of calculating lung volume may provide more accurate measurements as they require less patient cooperation [4,7,8]. Although CT-based methods provide reliable lung volume estimates that strongly correlate with those obtained by PFTs [9], they are technically demanding and involve extensive radiation exposure. Thus, there is an interest in estimating PFT values from measurements taken from routine radiographs, the gold-standard modality for scoliosis assessment for longitudinal studies due to lower radiation exposure and easy accessibility [10].

Previous studies on EOS patients have used simple linear regressions and correlations to predict PFT values from radiographic thoracospinal deformity parameters, reporting a wide range of precision (|*r*| = 0.11–0.72) [11–17]. Other studies utilized multiple regression to improve predictions, but the resulting precision of the models was poor (R^2^ = 0.19–0.38) [18,19]. In addition to poor precision, these prior studies included a maximum of only four radiographic parameters. Thus, the purpose of the current study was to develop regression models to predict PFT values with clinically useful precision in EOS patients using a more comprehensive list of radiographic thoracospinal deformity parameters. This analysis may not only yield predictive models for assessing pulmonary function during routine radiographic evaluations, but also quantify the relationships between thoracospinal deformity dimensions and pulmonary function in EOS patients to help guide surgical treatment.

## Materials and methods

### Study design

Preoperative full-length frontal and lateral spine radiographs and corresponding results of spirometry testing taken less than 90 days apart from 47 EOS patients (13 males/ 34 females; mean age: 9.8 ± 3.0 years; 31 idiopathic, 5 congenital, and 11 syndromic) were obtained retrospectively from the Pediatric Spine Study Group (PSSG), a multi-institutional EOS registry based in the United States. These patient data were fully deidentified and accessed for research purposes between August 2020 and April 2024. Percentage of predicted normal (i.e., percent predicted) forced vital capacity (%FVC) and forced expiratory volume in one second (%FEV_1_) were calculated at each pulmonary function laboratory using standard criteria, including patient age, height estimated by arm span or ulnar length, race, and sex. Using such arm-based measurements allows for more accurate percent predicted PFT calculations as it considers height loss secondary to scoliosis (i.e., it considers true patient height) [20]. Additionally, percent predicted instead of absolute PFT values were used to limit confounding factors in the analysis by controlling for inter-subject differences in age, height, and sex. The exclusion criteria were patients on ventilators or nighttime bilevel positive airway pressure (BiPAP)/ continuous positive airway pressure (CPAP) therapy and patients with neuromuscular scoliosis. This study protocol, including informed consent, parental permission forms, and assent forms, was reviewed and approved by the University of Utah Institutional Review Board (IRB # 00122910).

### Variables

**Fig 1:**
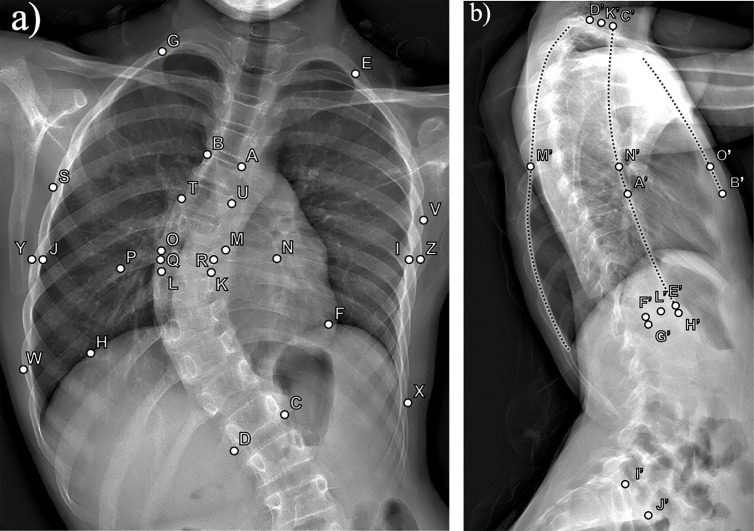
Anatomical landmark points used to define thoracospinal deformity parameters. Exemplar pair of a) frontal and b) sagittal plane radiographs with the anatomical landmark points used to calculate thoracospinal deformity parameters according to the formulas defined in Table 1.

Using a custom graphical user interface developed in MATLAB R2022a (The MathWorks Inc.) and pixel-to-millimeter scaling values obtained from the radiographic images, a total of 19 literature-based thoracospinal deformity parameters (Table 1) [3,21–26] were calculated from anatomical landmark points manually selected on frontal and lateral radiographs (Fig 1) by a trained observer. These parameters may be measured practically using common image processing software, but the custom program was employed simply to expedite the process of repeating measurements for all patient images. To validate measurement quality, a repeatability study was performed on a randomly selected 10% of the dataset, which, when rounded up, consisted of five patients. Using a two-way mixed-effects model for absolute agreement, the overall intraclass correlation coefficient (ICC) was 0.99. This value indicated excellent agreement [27] for repeated measurements made two months after the data collection period. Lastly, curvatures such as the major thoracic curve, thoracic kyphosis, and lumbar lordosis were assessed using the Cobb method, which involves measuring the angle between lines tangent to the superior and inferior endplates of the topmost and bottommost tilted vertebrae (i.e., cephalad and caudad end vertebrae) of a spinal curvature, respectively [28].

**Table 1:** Definitions and formulas for thoracospinal deformity parameters.

Parameter	Plane	Definition	Formula
Major thoracic curve	F	The Cobb angle of the largest spinal curve in the thoracic region	∠(AB,CD)
Cephalad end vertebral level	F	The thoracic vertebral level of the vertebra above the curve apex whose superior endplate is most tilted	
Apical vertebral level	F	The vertebra in a spinal curve that is most laterally deviated from the central sacral line (i.e., a vertical line that bisects the sacrum)	
Number of involved vertebrae	F	The number of vertebrae between the cephalad and caudad end vertebrae of a spinal curve	
Space available for the lung (SAFL)	F	Hemithoracic height is the distance between the midpoint of the cephalad-most rib to the midpoint of the ipsilateral hemidiaphragm. SAFL is the ratio of the concave divided by the convex hemithoracic heights expressed as a percentage [3]	$\begin{array}{c} \frac{EF}{GH}*100\\ \% \end{array}$
Lung height-width ratio (LHWr)	F	LHWr is the average hemithoracic heights (from SAFL) divided by the apical-level chest width which is a horizontal distance between the interior chest wall surfaces [21]	$\begin{array}{c} \frac{EF+GH}{2*IJ} \end{array}$
Apical rib vertebral angle difference (RVAD)	F	A perpendicular bisecting line with respect to the apical vertebral endplate is drawn. A line is drawn through the midpoints of the rib head and neck (just medial to the transition to the wider rib shaft) for both apical ribs. RVAD is the concave minus the convex angles formed between the rib lines and the perpendicular bisecting line [22]	∠(*MN*,*LK*⊥) − ∠(*OP*,*LK*⊥)
Apical vertebral body-rib ratio (AVBRr)	F	The horizontal distance between the lateral surface of the apical vertebra and the ipsilateral inner chest wall surface are measured bilaterally. AVBRr is the larger distance divided by the smaller distance [23]	$\begin{array}{c} \frac{JQ}{IR} \end{array}$
Parasol score	F	A thoracic width is a distance between the inner chest walls, and a hemithoracic width is the distance between a lateral vertebral surface to the ipsilateral chest wall. Parasol score is the ratio of the convex divided by the concave T6-level hemithoracic widths multiplied by the ratio of the T6-level thoracic width divided by the T12-level thoracic width [24]. All widths are measured perpendicular to the ribcage axis (i.e., a line that best approximates the rib cage orientation)	$\begin{array}{c} \frac{ST}{UV}*\frac{SV}{WX} \end{array}$
T6-level chest width	F	The distance between the inner chest walls at the T6 level measured perpendicular to the ribcage axis (from Parasol score)	SV
T12-level chest width	F	The distance between the inner chest walls at the T12 or final thoracic level measured perpendicular to the ribcage axis (from Parasol score)	WX
T6-level hemithorax asymmetry	F	The ratio of the concave divided by the convex hemithoracic widths at the T6 level measured perpendicular to the ribcage axis (from Parasol score) [25]	$\begin{array}{c} \frac{UV}{ST} \end{array}$
Sternovertebral distance	L	Horizontal distance between anterior apical vertebral surface and the internal sternal surface [26]. This distance is taken at the apical vertebral level	YZ
Frontosagittal index (FSI)	F & S	The ratio of the sternovertebral distance (in the frontal plane) divided by the horizontal distance between the anterior apical vertebral surface and the internal sternal surface (in the lateral plane) expressed as a percentage [26]. These distances are taken at the apical vertebral level	$\begin{array}{c} \frac{YZ}{A^{\prime }B^{\prime }}*100\\ \% \end{array}$
Thoracic kyphosis	S	The angle between the T1 and T12 superior and inferior endplates, respectively, using the Cobb method	∠(*C’D’*,*E’F’*)
Lumbar lordosis	S	The angle between the superior endplates of L1 and S1 using the Cobb method	∠(*G’H’*,*I’J’*)
Euclidean thoracic spine height	S	The Euclidean distance between the midpoints of the superior and inferior endplates of T1 and T12, respectively	KL
Rib hump depth index (RHDi)	S	Along a horizontal reference line drawn at the midpoint of the Euclidean thoracic spine height line, the distance between the interior chest wall of the convex rib hump and the anterior spinal column surface is measured. RHDi is the ratio of this distance divided by the Euclidean thoracic spine height [21]	$\begin{array}{c} \frac{MN}{KL} \end{array}$
Spinal intrusion ratio (SIr)	S	Along a horizontal reference line drawn at the midpoint of the Euclidean thoracic spine height line, SIr is the ratio of the distance between the interior chest wall of the convex rib hump and the anterior spinal column surface divided by the distance between the anterior spinal column surface and the internal sternal surface [21]	$\begin{array}{c} \frac{MN}{NO} \end{array}$

Thoracospinal deformity parameters measured on frontal (F) and sagittal (S) radiographic planes, along with their definitions and formulas. An exemplar pair of frontal and lateral radiographs with the anatomical landmark points used to calculate the deformity parameters is shown in Fig 1. Note: concave and convex sides, as well as the parameters of frontal spinal curvature like apical vertebra, are in reference to the major thoracic curve.

### Predictive model development and statistical analysis

The dataset was partitioned into model development and validation sets using a 90% (42 patients) and 10% (five patients) random split. Multiple linear regression (MLR) analysis was employed for model development in response to a preliminary analysis and the requirements of interpretability and statistical testing. Shapiro-Wilk tests were performed to confirm that %FVC and %FEV_1_ values were normally distributed, and initial scatter plots indicated linear relationships between these PFT values and each thoracospinal deformity parameter, which supported the use of MLR. Compared to non-linear and machine learning models, MLR analysis is less prone to overfitting in smaller datasets and provides both a clearer understanding and a statistical significance of each predictor’s influence, which are valuable in establishing evidence of the deformity parameters’ relationships with pulmonary function.

An exhaustive search algorithm developed in MATLAB was utilized to identify the optimal subset of thoracospinal deformity parameters that generated the best-fitting model for predicting the PFT values. For all MLR models that resulted from every combination of the 19 deformity parameters, the coefficients (β), standardized coefficients (std. β) used to ascertain the contribution of each predictor, *p* values, and variance inflation factors (VIFs) were calculated for each independent variable. Amongst MLR models with non-problematic collinearity, as indicated by VIFs < 10 [29], and statistically significant variables with *p* values < 0.05, the model with the highest adjusted coefficient of determination (adjusted R^2^) was selected. This analysis was performed separately for predicting %FVC and %FEV_1_ values.

For each final MLR model, the R^2^, standard error of the estimate (SEE), and *p* value were calculated, and actual versus predicted scatter plots with 95% prediction intervals were used to visually assess the goodness of fit and reliability of future predictions. To further assess multicollinearity, groups of highly correlated deformity parameters with |*r*| values > 0.8 [30] were identified beforehand using a correlation matrix, and the final MLR models were checked to ensure only one parameter from each group was present. Pearson correlation coefficients (*r*) and *p* values were also used to assess the strength and direction of the relationships between each deformity parameter and the PFT measurements. Normal expected cumulative probability-versus-observed cumulative probability plots (P-P plots) of regression standardized residuals were used to check the linear relationship assumption and that the residuals were approximately normally distributed, and scatter plots of standardized residuals versus standardized predicted values were used to check for normal distribution and homoscedasticity of the residuals. Durbin-Watson statistic values between 1.5 and 2.5 and maximum Cook’s distance values < 1 were used as thresholds to support that each observation was independent (i.e., no autocorrelation was present) and no influential data outliers were biasing the models, respectively. Lastly, the predictive accuracy was assessed on the separate validation dataset (*n *= 5) using the mean and standard deviation of the absolute errors, reported as mean absolute error (MAE) ± standard deviation. A Mann-Whitney U test was then used to detect significant differences in absolute error distributions between the model development and validation data, with *p* < 0.05 indicating statistical significance. All statistics were calculated using SPSS v28.0 (IBM Corp.).

## Results

Thoracospinal deformity parameter values for the 42 EOS patients in the model development set are summarized in Table 2. The bivariate relationships between deformity parameters and PFT measurements can be ascertained by the correlation coefficients found in Table 3. The following deformity parameters were found to have significant (*p* < 0.05) correlations with PFT measurements: major thoracic curve, space available for the lung (SAFL), apical vertebral body-rib ratio (AVBRr), parasol score, T6-level chest width, T12-level chest width, T6-level hemithorax asymmetry, frontosagittal index (FSI), thoracic kyphosis, and Euclidean thoracic spine height.

**Table 2:** Descriptive statistics for pulmonary function values and thoracospinal measurements.

Parameter	Median	First Quartile	Third Quartile	Minimum	Maximum
%FVC	79	66	92	28	122
%FEV_1_	73	58	84	26	120
Major thoracic curve (°)	65.27	53.03	74.87	31.13	89.37
Cephalad end vertebral level (thoracic level)	5	5	6	2	9
Apical vertebral level (thoracic level)	8	7	9	5	11
Number of involved vertebrae (vertebral levels)	5	4	6	2	9
Space available for the lung (SAFL) (%)	91.48	86.51	97.71	58.81	107.76
Lung height-width ratio (LHWr)	0.78	0.73	0.84	0.58	0.98
Apical rib vertebral angle difference (RVAD) (°)	19.99	6.96	39.25	−12.97	84.88
Apical vertebral body-rib ratio (AVBRr)	1.90	1.70	2.47	1.05	10.17
Parasol score	0.59	0.46	0.75	0.27	0.98
T6-level chest width (cm)	18.56	16.94	21.73	12.89	25.19
T12-level chest width (cm)	20.02	18.65	23.24	14.32	28.45
T6-level hemithorax asymmetry	1.60	1.30	1.94	0.90	3.32
Sternovertebral distance (cm)	7.57	6.15	8.56	4.18	12.80
Frontosagittal index (FSI) (%)	42.26	33.23	49.10	23.94	78.19
Thoracic kyphosis (°)	30.16	15.19	39.60	−0.17	75.60
Lumbar lordosis (°)	55.90	50.21	68.30	23.63	85.80
Euclidean thoracic spine height (cm)	19.91	16.75	22.58	11.14	27.48
Rib hump depth index (RHDi)	0.29	0.26	0.34	0.20	0.60
Spinal intrusion ratio (SIr)	0.73	0.59	1.04	0.28	1.80

Descriptive statistics of percent predicted forced vital capacity (%FVC) values, forced expiratory volume in one second (%FEV_1_) values, and the measured thoracospinal deformity parameters (*n* = 42).

**Table 3:** Correlations between thoracospinal deformity parameters and pulmonary function values.

Parameter	%FVC	%FEV_1_
Major thoracic curve (°)	−0.498^**^	−0.515^**^
Cephalad end vertebral level (thoracic level)	0.163	0.161
Apical vertebral level (thoracic level)	0.117	0.074
Number of involved vertebrae (thoracic level)	−0.162	−0.247
Space available for the lung (SAFL) (%)	0.426^**^	0.398^**^
Lung height-width ratio (LHWr)	−0.031	0.021
Apical rib vertebral angle difference (RVAD) (°)	−0.144	−0.194
Apical vertebral body-rib ratio (AVBRr)	−0.409^**^	−0.450^**^
Parasol score	0.455^**^	0.492^**^
T6-level chest width (cm)	0.486^**^	0.492^**^
T12-level chest width (cm)	0.482^**^	0.472^**^
T6-level hemithorax asymmetry	−0.524^**^	−0.549^**^
Sternovertebral distance (cm)	−0.028	−0.073
Frontosagittal index (FSI) (%)	−0.297	−0.356^*^
Thoracic kyphosis (°)	−0.274	−0.307^*^
Lumbar lordosis (°)	−0.073	−0.058
Euclidean thoracic spine height (cm)	0.406^**^	0.433^**^
Rib hump depth index (RHDi)	−0.252	−0.269
Spinal intrusion ratio (SIr)	−0.012	0.018

Pearson correlation coefficients (*r*) for thoracospinal deformity parameters with percent predicted forced vital capacity (%FVC) and forced expiratory volume in one second (%FEV_1_) values (*n *= 42). *p < 0.05, **p < 0.01.

**Fig 2:**
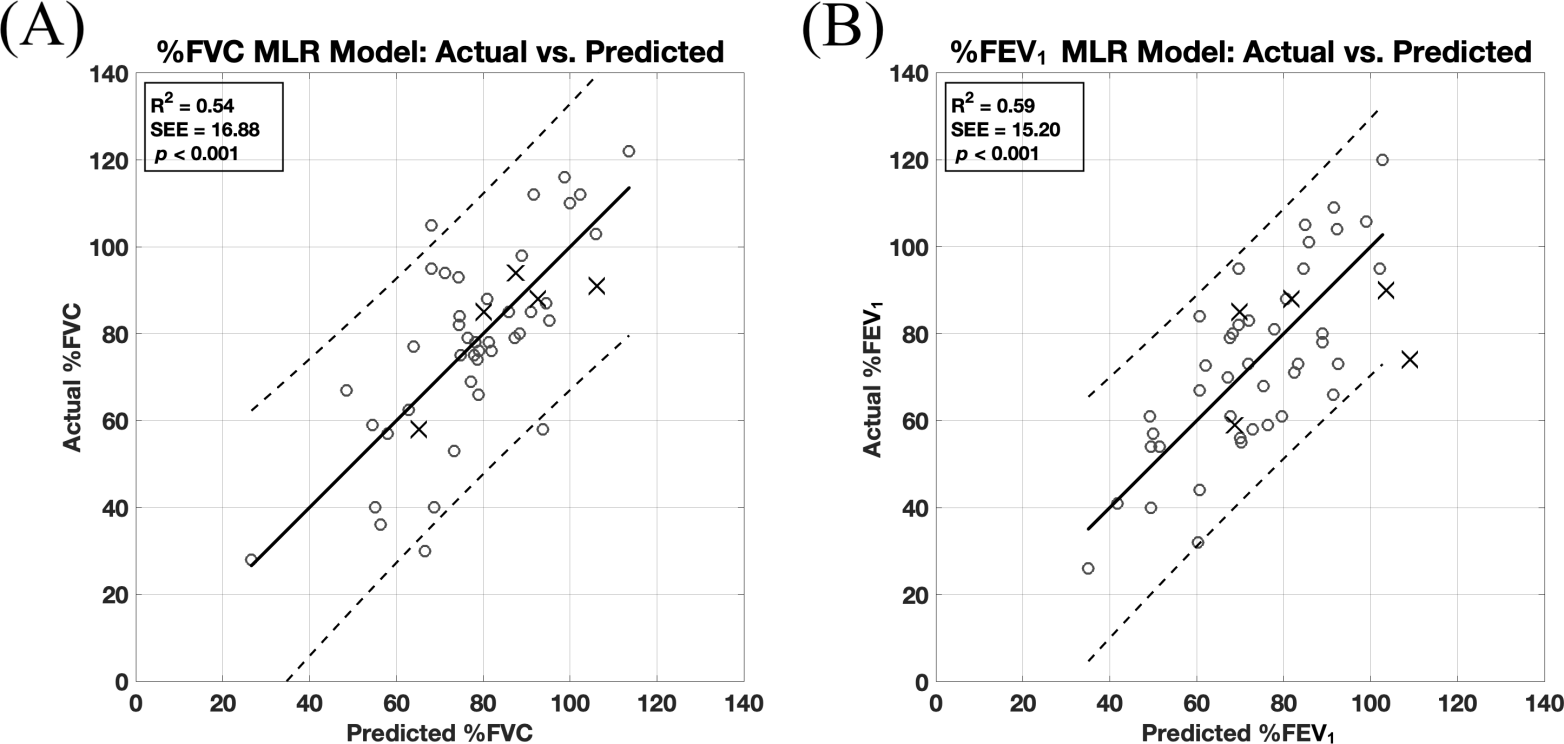
Actual versus predicted pulmonary function values. Actual values of percent predicted (A) forced vital capacity (%FVC) and (B) forced expiratory volume in one second (%FEV_1_) versus their corresponding predicted values by multiple linear regression (MLR) models using thoracospinal deformity parameters. The circles (○) and X’s (×) represent the data points used for model development (*n* = 42) and validation (*n* = 5), respectively. The coefficient of determination (R^2^), standard error of the estimate (SEE), and *p* values are reported. For each model, the line of best fit and the 95% prediction intervals are shown as solid and dotted lines, respectively.

Each MLR model (Table 4) was statistically significant (*p* < 0.001) and satisfied all assumption criteria, including the exclusion of highly correlated deformity parameters, which were identified from the correlation matrix (S1 Table). The model for predicting %FVC had an R^2^ of 0.54 and an SEE of 16.88%, and the model for %FEV_1_ had an R^2^ of 0.59 and an SEE of 15.20% (Fig 2). Notably, in the exhaustive search analysis, these models with the highest adjusted R^2^ were also the ones with the highest R^2^ values. The following thoracospinal deformity parameters exerted significant influence on predicting %FVC and %FEV_1_: spinal intrusion ratio (SIr), T6-level chest width, sternovertebral distance, rib hump depth index (RHDi), and thoracic kyphosis. For the %FVC model, an additional significant parameter was T6-level hemithorax asymmetry, and for the %FEV_1_ model, cephalad end vertebral level and major thoracic curve were additional significant parameters. The contribution of each predictor can be ascertained from the absolute values of Standardized β (Table 4). Lastly, the MAEs from the validation tests for the %FVC and %FEV_1_ models were 8% ± 4% and 16% ± 11%, respectively, and no significant differences were detected between the distributions of these absolute errors and those of the corresponding model residuals.

**Table 4:** Regression models predicting pulmonary function from thoracospinal parameters.

Pulmonary Function Test Measurement (PFTM)	Thoracospinal Deformity Parameter (TDP)	β	Std. β	*p*	VIF
%FVC	(Constant)	112.55		0.003	
	Spinal intrusion ratio (SIr)	−81.39	−1.15	0.001	7.77
	T6-level chest width (cm)	4.52	0.62	0.002	2.61
	Sternovertebral distance (cm)	−7.03	−0.56	0.025	4.30
	Rib hump depth index (RHDi)	129.24	0.40	0.031	2.34
	Thoracic kyphosis (°)	−0.50	−0.40	0.042	2.64
	T6-level hemithorax asymmetry	−15.34	−0.38	0.012	1.52
%FEV_1_	(Constant)	78.51		0.036	
	Spinal intrusion ratio (SIr)	−74.30	−1.12	0.002	9.25
	Thoracic kyphosis (°)	−0.71	−0.60	0.004	3.08
	T6-level chest width (cm)	4.01	0.58	0.006	3.27
	Sternovertebral distance (cm)	−6.12	−0.52	0.044	5.04
	Rib hump depth index (RHDi)	127.88	0.42	0.027	2.68
	Cephalad end vertebral level (thoracic level)	5.78	0.38	0.009	1.49
	Major thoracic curve (°)	−0.39	−0.29	0.049	1.65

Multiple linear regression (MLR) models used to estimate percent predicted forced vital capacity (%FVC) and forced expiratory volume in one second (%FEV_1_) values using thoracospinal deformity parameters ordered in terms of contribution (*n* = 42). The MLR models are in the form: PFTM = Σ(βi*TDPi) + Constant, where PFTM is a pulmonary function test measurement, βi is a coefficient, and TDPi is a thoracospinal deformity parameter. For each deformity parameter, the standardized coefficient (Std. β), *p* value, and variance inflation factor (VIF) are also reported.

## Discussion

### Relationships between thoracospinal deformity severity and pulmonary function deficit

Given the limited number of EOS studies, findings from adolescent idiopathic scoliosis (AIS) studies will also be referenced to enrich the broader discussion of the impact of skeletal deformity on pulmonary function deficit in children with scoliosis. The directionalities of the statistically significant correlations between the thoracospinal deformity parameters and %FVC and %FEV_1_ values (Table 3) are in general agreement with previously reported relationships between deformity parameters and PFT values in scoliotic children and even adult idiopathic scoliosis [11,14,17,18,21,25,31–38]. Most notably, the current study supports the thoroughly studied negative correlation between the major thoracic curve and PFT values [11,14–16,19,21,31,34,36,39–41]. However, there are seemingly contradictory reports on the relationship between pulmonary function and thoracic kyphosis. In agreement with a study on AIS [33], thoracic kyphosis was negatively correlated with PFT values, which may be related to less available lung space due to the associated thoracic height loss. Conversely, others have found a significant positive correlation to exist between thoracic kyphosis and PFT values in AIS patients [19,21,40]. The decrease in kyphosis may result in diminished lung space through decreased anteroposterior chest diameter. These seemingly conflicting results may be explained by the findings of Dreimann et al. [32] that both lower- and upper-spectrum deviations from normal sagittal spinal curvature (i.e., hypo- or hyperkyphosis) may lead to pulmonary impairment in children with scoliosis. Overall, the statistically significant relationships quantified in the current study support that a larger and more cephalad (i.e., closer to the lung space) coronal spinal curvature, and a thoracic cage that is less symmetric, has a larger rib hump, and is reduced in volume (i.e., reduced height, width, and/or depth) are characteristics most associated with diminished lung function in EOS patients. Additionally, the finding that the directionality of these relationships does not differ between %FVC and %FEV_1_ was expected as it was previously reported that a strong, positive linear mathematical relationship (*r *= 0.96) exists between FVC and FEV_1_ [42]. It is also for this reason that the strengths of the bivariate correlations and the goodness of fit for the MLR models (i.e., R^2^ = 0.54 and R^2^ = 0.59) are similar between %FVC and %FEV_1_.

Considering that the deformity parameters collected are taken with respect to the concave versus convex sidedness but are agnostic to whether the spinal curve is left or right, an additional analysis was performed to determine if there was a significant difference in PFT values between left- and right-sided curve patient cohorts. After a two-sample t-test confirmed that major thoracic curve magnitudes were not significantly different between the two cohorts, a subsequent t-test then supported that no differences in either %FVC or %FEV_1_ were present between left- and right-sided cohorts.

### Efficacy of predicting PFT values from radiographic deformity parameters using multiple linear regression

A limited number of studies have estimated PFT values from radiographic thoracospinal deformity parameters in EOS patients. Some have utilized simple linear regressions or correlations and found poor to moderate predictions of pre- and postoperative %PFT using Cobb angles (|*r*| = 0.11–0.72) [11–16] and thoracic spine height (*r* ≤ 0.63) [17]. However, these reported relationship strengths in two of these studies [13,15] may be diminished partly due to the inclusion of the neuromuscular etiology, where respiratory muscle weakness is a confounding variable. Only two reports have utilized multivariable modeling. In a study of 121 EOS patients with idiopathic, congenital, syndromic, and neuromuscular etiologies, Glotzbecker et al. [18] considered pelvic inlet width, thoracic spine height, T1-S1 height, and coronal chest width measurements and found only the following two parameters to be significant predictors of %PFT: pelvic inlet width (|*r*| ≤ 0.42 for %FVC, %FEV_1_, and percent predicted total lung capacity (%TLC)) and thoracic spine height normalized to pelvic inlet width (*r* = 0.32 for %FEV_1_). Their subsequent MLR analysis revealed that the normalized thoracic spine height was the only significant predictor of %FVC and %FEV_1_ and that no thoracic dimension was a significant predictive factor for %TLC. However, pairs of measurements successfully formed MLR models for absolute PFT values (R^2^ = 0.26 for FVC, R^2^ = 0.21 for FEV_1_, and R^2^ = 0.38 for TLC). In short, it was concluded that thoracic dimensions are weak predictors of PFT values. In the second study, Johnston et al. [19] used data from 858 AIS patients – 9.1% of which were classified under juvenile idiopathic scoliosis where onset was before 10 years of age – to develop an MLR model consisting of major thoracic curve, thoracic kyphosis, and apical vertebral rotation but was only able to explain 18.5% of the variance in FVC or FEV_1_.

Compared to these prior efforts, the current study considered the most comprehensive set of radiographic thoracospinal deformity parameters to predict PFT values in preoperative EOS patients, and successfully generated regression models that can predict %FVC (R^2^ = 0.54) and %FEV_1_ (R^2^ = 0.59) with the highest reported levels of precision. Also, the predictive performance of the models held for new data, as no significant difference was found between model residual errors and validation test errors. This is further supported by the good agreement between model SEE and validation MAE values. Despite this preliminary indication of generalizability, the predictions that these MLR models produce may have limited clinical utility. Due to wide prediction intervals, predicting pulmonary deficit (i.e., actual %PFT values below 80%) with a 95% confidence can only be done with model-predicted values near the peripheral low end of the observed range. For example, predicting actual %FEV_1_ values below 80% with 95% confidence requires predicted values from the MLR model to be below 51%. Despite such limitations, the key subset of thoracospinal deformity parameters selected for the MLR models using the exhaustive search algorithm still bears clinical relevance as they most affect pulmonary function in EOS patients as compared to the rest of the parameters considered. Specifically, the ordered list of deformity parameters in terms of their relative contributions to predicting PFT values in the MLR models may aid in surgical planning by prioritizing the correction of thoracospinal dimensions that contribute most to pulmonary function impairment. Additionally, it is important to note that the signs of the coefficients (i.e., positive or negative) for some deformity parameters in MLR models contradict their true bivariate relationships with PFT values found in Table 3. Such changes in directionality may occur when the independent variables of an MLR model are unstandardized (i.e., were not all centered and scaled in the same manner), as is the case herein, where deformity parameters were purposefully expressed in their different original units and scales for ease of making predictions using the MLR models.

### Limitations

The retrospective radiographic images used in the current study were obtained from a multicenter registry and had heterogeneous resolution. Consequently, this limited the types of thoracospinal deformity parameters included for analysis, because measurements that require more granular radiographic details could not be precisely measured. For example, pedicle projection positions required to quantify vertebral rotation [34,36,43,44], a typical phenomenon that is coupled with coronal curvatures in scoliotic deformity [45,46], could not be measured precisely on the radiographs. However, such measurements have low reliability [40] and are known to significantly correlate with the major thoracic curve [33], which was included in the current study. Other deformity parameters could not have been measured from retrospective imaging data, such as spinal curve flexibility, which has been reported to influence pulmonary function in AIS and adult idiopathic scoliosis [32,33,40,47].

The MLR models developed herein are only applicable to EOS patients, as the age of scoliosis onset is a key prognostic factor and influences the relationships between deformity parameters and PFT values [16]. Additionally, the inclusion of a heterogeneous cohort of EOS etiologies (i.e., idiopathic, congenital, and syndromic) in this study may have unknown effects, as there is limited knowledge on how lung growth and function differ between each etiology. The mathematical relationships developed herein may also be limited by suboptimal patient compliance with breathing instructions during radiographic image acquisition and PFT testing, as well as by the well-documented variable accuracy of PFT related to intra-subject variability and using percentage of predicted normal values [5,6,48,49].

The unexplained variance in the %PFT values may also be attributed to the limitation of using 2D static deformity measurements to predict dynamic PFT values. For this reason, it is expected that more precise predictions may be made of static CT-derived lung volumes in EOS patients using the radiographic deformity parameters reported herein, similar to the highly precise regression models developed for normative pediatric subjects using 2D radiographic lung and diaphragm measurements [50]. However, CT-based methods are technically demanding and involve extensive radiation exposure, which limits the ability to acquire such data serially, and the resulting static lung volumes would also be limited in clinical utility as they do not consider the altered chest and diaphragm breathing dynamics found in EOS deformities. So, future work may include predicting PFT values in EOS patients from dynamic magnetic resonance imaging [51].

The small sample size is another limitation of this study and is attributed to the rarity of the disease and the difficulty in obtaining reliable PFT values from young patients and corresponding radiographs of sufficient image quality. Despite the relatively small sample size, the risk of predictive model overfitting was mitigated by the exhaustive feature selection method, multicollinearity assessment, and validation testing. With larger sample sizes, more precise predictions of PFT values from radiographic deformity parameters may be achieved using machine learning methods [52]. Such methods may also be applied to completely automate the extraction of deformity parameters from radiographs as well as the classification of deformity [53]. Additionally, along with spinal growth models [54–56] and reference data [57–61], developing models to predict postoperative PFT values would further aid in clinical decision making and grading surgical outcomes. It is expected that radiographic deformity parameters will improve after surgery, and consequently, so will PFT values. However, it has been reported that postoperative changes in deformity parameters such as major thoracic curve do not correlate with the resulting improvements in pulmonary function [62]. Future work can focus on elucidating the relationships between improvements in the deformity parameters used herein and the PFT values in postoperative data.

## Conclusions

In the effort to predict PFT values in preoperative EOS patients directly from routine radiographs, the current study has considered the largest variety of thoracospinal deformity parameters to date. The regression models developed herein can predict %FVC (R^2^ = 0.54) and %FEV_1_ (R^2^ = 0.59) with the highest reported level of precision. Additionally, the relative contributions of each deformity parameter to predicting the PFT values provide quantitative metrics to guide surgical treatment.

## Supporting information

S1 Table Correlation matrix of thoracospinal deformity parameters. Correlation matrix containing the Pearson correlation coefficients (*r*) for bivariate relationships amongst thoracospinal deformity parameters (*n* = 42). Three groups of parameters that are highly correlated (|*r*| > 0.8) are 1) apical vertebral level and cephalad end vertebral level, 2) parasol score and T6-level hemithorax asymmetry, and 3) T6-level chest width, T12-level chest width, and Euclidean thoracic spine height. *p < 0.05, **p < 0.01.
(DOCX)
