# Supplementary material for: Predicting pulmonary function using thoracic deformity parameters in early onset scoliosis patients
Source: PLoS One. 2025 Jul 31;20(7):e0329199. doi: 10.1371/journal.pone.0329199 (PMC12312942; doi:10.1371/journal.pone.0329199)
Supplement: S1 Table — Correlation matrix containing the Pearson correlation coefficients (r) for bivariate relationships amongst thoracospinal deformity parameters (n = 42). Three groups of parameters that are highly correlated (|r| > 0.8) are 1) apical vertebral level and cephalad end vertebral level, 2) parasol score and T6-level hemithorax asymmetry, and 3) T6-level chest width, T12-level chest width, and Euclidean thoracic spine height. *p < 0.05, **p < 0.01. (DOCX) [file pone.0329199.s001.docx]

| **Spinal intrusion ratio (SIr)** | -0.012 | 0.018 | 0.017 | -0.134 | -0.151 | 0.146 | 0.173 | 0.304^*^ | -0.263 | -0.359^*^ | 0.097 | 0.288 | 0.187 | -0.155 | -0.787^**^ | -0.768^**^ | -0.720^**^ | -0.501^**^ | 0.334^*^ | 0.447^**^ | . |
| --- | --- | --- | --- | --- | --- | --- | --- | --- | --- | --- | --- | --- | --- | --- | --- | --- | --- | --- | --- | --- | --- |
| **Rib hump depth index (RHDi)** | -0.252 | -0.269 | 0.273 | -0.343^*^ | -0.242 | 0.417^**^ | -0.311^*^ | 0.127 | 0.213 | 0.127 | -0.428^**^ | -0.323^*^ | -0.257 | 0.371^*^ | -0.311^*^ | -0.100 | -0.082 | 0.117 | -0.422^**^ | . | 0.447^**^ |
| **Euclidean thoracic spine height (cm)** | 0.406^**^ | 0.433^**^ | -0.383^*^ | -0.025 | -0.168 | -0.228 | 0.285 | 0.223 | -0.436^**^ | -0.559^**^ | 0.375^*^ | 0.916^**^ | 0.845^**^ | -0.405^**^ | -0.021 | -0.543^**^ | -0.578^**^ | -0.443^**^ | . | -0.422^**^ | 0.334^*^ |
| **Lumbar lordosis (°)** | -0.073 | -0.058 | 0.109 | 0.050 | 0.103 | 0.047 | -0.413^**^ | -0.412^**^ | 0.316^*^ | 0.385^*^ | -0.170 | -0.355^*^ | -0.278 | 0.163 | 0.453^**^ | 0.565^**^ | 0.574^**^ | . | -0.443^**^ | 0.117 | -0.501^**^ |
| **Thoracic kyphosis (°)** | -0.274 | -0.307^*^ | 0.136 | 0.263 | 0.311^*^ | 0.106 | -0.399^**^ | -0.523^**^ | 0.385^*^ | 0.589^**^ | -0.121 | -0.482^**^ | -0.473^**^ | 0.249 | 0.504^**^ | 0.717^**^ | . | 0.574^**^ | -0.578^**^ | -0.082 | -0.720^**^ |
| **Frontosagittal index (FSI) (%)** | -0.297 | -0.356^*^ | 0.357^*^ | 0.120 | 0.235 | -0.054 | -0.358^*^ | -0.305^*^ | 0.362^*^ | 0.601^**^ | -0.351^*^ | -0.549^**^ | -0.450^**^ | 0.405^**^ | 0.760^**^ | . | 0.717^**^ | 0.565^**^ | -0.543^**^ | -0.100 | -0.768^**^ |
| **Sternovertebral distance (cm)** | -0.028 | -0.073 | 0.068 | -0.024 | -0.048 | -0.226 | -0.247 | -0.261 | 0.144 | 0.198 | -0.183 | 0.040 | 0.175 | 0.204 | . | 0.760^**^ | 0.504^**^ | 0.453^**^ | -0.021 | -0.311^*^ | -0.787^**^ |
| **T6-level hemithorax asymmetry** | -0.524^**^ | -0.549^**^ | 0.367^*^ | -0.606^**^ | -0.406^**^ | 0.278 | -0.594^**^ | 0.180 | 0.408^**^ | 0.556^**^ | -0.941^**^ | -0.419^**^ | -0.321^*^ | . | 0.204 | 0.405^**^ | 0.249 | 0.163 | -0.405^**^ | 0.371^*^ | -0.155 |
| **T12-level chest width (cm)** | 0.482^**^ | 0.472^**^ | -0.410^**^ | -0.161 | -0.281 | -0.205 | 0.197 | 0.092 | -0.286 | -0.588^**^ | 0.258 | 0.935^**^ | . | -0.321^*^ | 0.175 | -0.450^**^ | -0.473^**^ | -0.278 | 0.845^**^ | -0.257 | 0.187 |
| **T6-level chest width (cm)** | 0.486^**^ | 0.492^**^ | -0.431^**^ | -0.070 | -0.194 | -0.196 | 0.299 | 0.079 | -0.349^*^ | -0.608^**^ | 0.402^**^ | . | 0.935^**^ | -0.419^**^ | 0.040 | -0.549^**^ | -0.482^**^ | -0.355^*^ | 0.916^**^ | -0.323^*^ | 0.288 |
| **Parasol score** | 0.455^**^ | 0.492^**^ | -0.348^*^ | 0.620^**^ | 0.441^**^ | -0.225 | 0.547^**^ | -0.173 | -0.381^*^ | -0.409^**^ | . | 0.402^**^ | 0.258 | -0.941^**^ | -0.183 | -0.351^*^ | -0.121 | -0.170 | 0.375^*^ | -0.428^**^ | 0.097 |
| **Apical vertebral body-rib ratio (AVBRr)** | -0.409^**^ | -0.450^**^ | 0.497^**^ | 0.174 | 0.291 | 0.163 | -0.463^**^ | -0.229 | 0.402^**^ | . | -0.409^**^ | -0.608^**^ | -0.588^**^ | 0.556^**^ | 0.198 | 0.601^**^ | 0.589^**^ | 0.385^*^ | -0.559^**^ | 0.127 | -0.359^*^ |
| **Apical rib vertebral angle difference (RVAD) (°)** | -0.144 | -0.194 | 0.427^**^ | -0.008 | 0.176 | 0.328^*^ | -0.271 | -0.290 | . | 0.402^**^ | -0.381^*^ | -0.349^*^ | -0.286 | 0.408^**^ | 0.144 | 0.362^*^ | 0.385^*^ | 0.316^*^ | -0.436^**^ | 0.213 | -0.263 |
| **Lung height-width ratio (LHWr)** | -0.031 | 0.021 | -0.094 | -0.438^**^ | -0.470^**^ | 0.037 | 0.022 | . | -0.290 | -0.229 | -0.173 | 0.079 | 0.092 | 0.180 | -0.261 | -0.305^*^ | -0.523^**^ | -0.412^**^ | 0.223 | 0.127 | 0.304^*^ |
| **Space available for the lung (SAFL) (%)** | 0.426^**^ | 0.398^**^ | -0.349^*^ | 0.290 | 0.155 | -0.343^*^ | . | 0.022 | -0.271 | -0.463^**^ | 0.547^**^ | 0.299 | 0.197 | -0.594^**^ | -0.247 | -0.358^*^ | -0.399^**^ | -0.413^**^ | 0.285 | -0.311^*^ | 0.173 |
| **Number of involved vertebrae (thoracic level)** | -0.162 | -0.247 | 0.073 | -0.354^*^ | 0.058 | . | -0.343^*^ | 0.037 | 0.328^*^ | 0.163 | -0.225 | -0.196 | -0.205 | 0.278 | -0.226 | -0.054 | 0.106 | 0.047 | -0.228 | 0.417^**^ | 0.146 |
| **Apical vertebral level (thoracic level)** | 0.117 | 0.074 | 0.205 | 0.795^**^ | . | 0.058 | 0.155 | -0.470^**^ | 0.176 | 0.291 | 0.441^**^ | -0.194 | -0.281 | -0.406^**^ | -0.048 | 0.235 | 0.311^*^ | 0.103 | -0.168 | -0.242 | -0.151 |
| **Cephalad end vertebral level (thoracic level)** | 0.163 | 0.161 | 0.127 | . | 0.795^**^ | -0.354^*^ | 0.290 | -0.438^**^ | -0.008 | 0.174 | 0.620^**^ | -0.070 | -0.161 | -0.606^**^ | -0.024 | 0.120 | 0.263 | 0.050 | -0.025 | -0.343^*^ | -0.134 |
| **Major thoracic curve (°)** | -0.498^**^ | -0.515^**^ | . | 0.127 | 0.205 | 0.073 | -0.349^*^ | -0.094 | 0.427^**^ | 0.497^**^ | -0.348^*^ | -0.431^**^ | -0.410^**^ | 0.367^*^ | 0.068 | 0.357^*^ | 0.136 | 0.109 | -0.383^*^ | 0.273 | 0.017 |
| **%FEV_1_** | 0.929^**^ | . | -0.515^**^ | 0.161 | 0.074 | -0.247 | 0.398^**^ | 0.021 | -0.194 | -0.450^**^ | 0.492^**^ | 0.492^**^ | 0.472^**^ | -0.549^**^ | -0.073 | -0.356^*^ | -0.307^*^ | -0.058 | 0.433^**^ | -0.269 | 0.018 |
| **%FVC** | . | 0.929^**^ | -0.498^**^ | 0.163 | 0.117 | -0.162 | 0.426^**^ | -0.031 | -0.144 | -0.409^**^ | 0.455^**^ | 0.486^**^ | 0.482^**^ | -0.524^**^ | -0.028 | -0.297 | -0.274 | -0.073 | 0.406^**^ | -0.252 | -0.012 |
| **Parameter** | **%FVC** | **%FEV_1_** | **Major thoracic curve (°)** | **Cephalad end vertebral level (thoracic level)** | **Apical vertebral level (thoracic level)** | **Number of involved vertebrae (thoracic level)** | **Space available for the lung (SAFL) (%)** | **Lung height-width ratio (LHWr)** | **Apical rib vertebral angle difference (RVAD) (°)** | **Apical vertebral body-rib ratio (AVBRr)** | **Parasol score** | **T6-level chest width (cm)** | **T12-level chest width (cm)** | **T6-level hemithorax asymmetry** | **Sternovertebral distance (cm)** | **Frontosagittal index (FSI) (%)** | **Thoracic kyphosis (°)** | **Lumbar lordosis (°)** | **Euclidean thoracic spine height (cm)** | **Rib hump depth index (RHDi)** | **Spinal intrusion ratio (SIr)** |
